# The Role of Prehabilitation in Modern Esophagogastric Cancer Surgery: A Comprehensive Review

**DOI:** 10.3390/cancers14092096

**Published:** 2022-04-22

**Authors:** Augustinas Bausys, Morta Mazeikaite, Klaudija Bickaite, Bernardas Bausys, Rimantas Bausys, Kestutis Strupas

**Affiliations:** 1 Clinic of Gastroenterology, Nephrourology, and Surgery, Faculty of Medicine, Institute of Clinical Medicine, Vilnius University, 03101 Vilnius, Lithuania; bausys@gmail.com (R.B.); kestutis.strupas@santa.lt (K.S.); 2 Department of Abdominal Surgery and Oncology, National Cancer Institute, 08660 Vilnius, Lithuania; 3 Centre for Visceral Medicine and Translational Research, Faculty of Medicine, Institute of Clinical Medicine, Vilnius University, 03101 Vilnius, Lithuania; klaudia.bickaite@gmail.com (K.B.); barniux@gmail.com (B.B.); 4 Department of Rehabilitation, Physical and Sports Medicine, Faculty of Medicine, Institute of Health Sciences, Vilnius University, 03101 Vilnius, Lithuania; mazeikaite.morta@gmail.com

**Keywords:** esophageal cancer, gastric cancer, esophagectomy, gastrectomy, prehabilitation, exercise

## Abstract

**Simple Summary:**

Surgery is the only potentially curative treatment option for esophagogastric cancer. Although esophagectomy/gastrectomy remains associated with major surgical trauma and significant morbidity. Prehabilitation has emerged as a novel strategy to improve postoperative outcomes by preparing patients for a surgery-associated physiological challenge. We discuss current knowledge and the results of studies on the role of prehabilitation in esophagogastric cancer surgery.

**Abstract:**

Esophagogastric cancer is among the most common malignancies worldwide. Surgery with or without neoadjuvant therapy is the only potentially curative treatment option. Although esophagogastric resections remain associated with major surgical trauma and significant postoperative morbidity. Prehabilitation has emerged as a novel strategy to improve clinical outcomes by optimizing physical and psychological status before major surgery through exercise and nutritional and psychological interventions. Current prehabilitation programs may be unimodal, including only one intervention, or multimodal, combining the benefits of different types of interventions. However, it still is an investigational treatment option mostly limited to clinical trials. In this comprehensive review, we summarize the current evidence for the role of prehabilitation in modern esophagogastric cancer surgery. The available studies are very heterogeneous in design, type of interventions, and measured outcomes. Yet, all of them confirm at least some positive effects of prehabilitation in terms of improved physical performance, nutritional status, quality of life, or even reduced postoperative morbidity. However, the optimal interventions for prehabilitation remain unclear; thus, they cannot be standardized and widely adopted. Future studies on multimodal prehabilitation are necessary to develop optimal programs for patients with esophagogastric cancer.

## 1. Introduction

Esophagogastric cancer (esophageal and gastric cancer; EGC) is among the most common malignancies worldwide, with over 1.6 million new cases and 1.2 million deaths annually [1,2,3]. Surgery is the main and only curative treatment option [4,5]. However, gastric and esophageal resections remain associated with high postoperative morbidity and mortality rates [4,5,6]. Current evidence indicates the benefits of neoadjuvant chemo(radio)therapy [7,8,9]. Preoperative cytotoxic treatment improves oncological outcomes, but impairs patients’ physical and nutritional status, promotes sarcopenia, and decreases physiological reserve, thus further increasing the surgery-related risk [4,10,11,12]. Consequently, there is a need for novel strategies to improve EGC surgery outcomes.

Recently, prehabilitation has emerged as a way to prepare a patient for major surgery. As it is a relatively new concept in surgical oncology, definitions of prehabilitation still vary. They consistently state that it is a pre-emptive preparation of a patient to reduce risks and enhance recovery after a stressful event. Prehabilitation has significantly reduced postoperative morbidity in some high-risk patients undergoing major abdominal surgery [13]. Additionally, it reduces systemic inflammation [14], attenuates chemotherapy-induced toxicity [15], modulates several host- and tumor-related pathways during standard chemotherapy [15], and may even promote tumor regression following neoadjuvant therapy [16]. Current studies on prehabilitation are very heterogenous in a perioperative care pathway and measured outcomes. Moreover, some studies show controversial results, as prehabilitation has no benefit in frail patients undergoing minimally invasive colorectal cancer surgery [17]. Therefore, the role of prehabilitation in modern EGC surgery remains unclear. This review aims to comprehensively overview the current evidence for prehabilitation in patients undergoing major esophagogastric resections for cancer.

## 2. Literature Search Strategy

**Figure 1:**
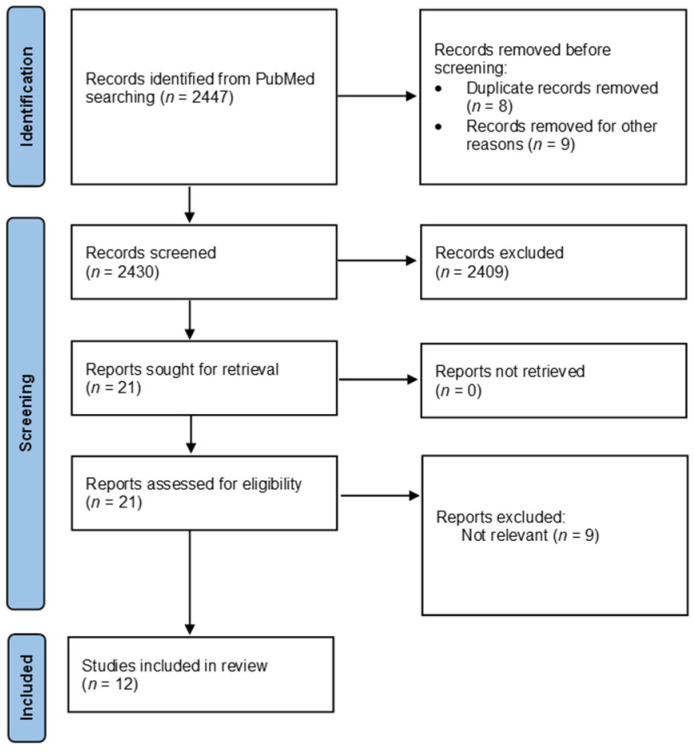
Literature search flow diagram.

A comprehensive literature search was conducted using the PubMed database last on 1 December 2021. The search term we used was ‘prehabilitation’ OR ‘exercise’ OR ‘nutritional support’ OR ‘psychological support’ AND ‘esophageal cancer’ OR ‘gastric cancer”. Time restrictions for publications were not used. Only manuscripts published in the English language were reviewed. Two independent reviewers (A.B. and K.B.) reviewed all titles and abstracts to identify clinical studies investigating prehabilitation in EGC patients. Full-text articles were retrieved if relevant abstracts were identified (Figure 1). An additional manual search of the reference lists was performed to ensure the comprehensive literature search procedure. The quality of evidence provided by each study was evaluated using the Jadad [18] and the Newcastle–Ottawa [19] scales for randomized and non-randomized studies, respectively.

## 3. The Current Concept of Prehabilitation in Esophagogastric Cancer Surgery

Current definitions of prehabilitation vary but consistently state that it is a pre-emptive preparation of a patient to reduce risks and enhance recovery after a stressful event. EGC surgery is an ideal example of a stressor because of extensive surgical trauma, physiological consequences of previous cytotoxic treatments, and psychological distress. These factors interact with the burden of cancer, which includes impaired nutritional and physiological reserves due to cachexia, malnutrition, and sarcopenia. The preoperative period constitutes a unique opportunity to prepare the patient for these challenges because most are highly motivated to change behavior for perioperative benefits [20]. Contemporary prehabilitation programs may include one (unimodal) or several (multimodal) interventions aiming to correct modifiable risk factors, promote a patient’s physical activity, optimize nutritional status, and intervene in psychological wellbeing. There is no consensus on the optimal design of a prehabilitation program; thus, different approaches have been investigated (Table 1).

Among them, there are 5 randomized control trials (RCTs) [21,22,23,24,26], 4 pilot studies [25,29,30,31], 2 non-randomized control trials [27,28], and 1 matched-pair analysis [32]. Despite the fact that all studies focused on prehabilitation for EGC surgery, they are heterogeneous in applied interventions and measured outcomes. Table 2 and Table 3 show the structure of prehabilitation programs and their impact on clinical outcomes.

### 3.1. Exercise Interventions in Unimodal and Multimodal Prehabilitation Programs

Exercise has obvious and indisputable benefits on individuals’ health, including those who have cancer. Physical activity increases fitness levels and physical functioning. It also decreases cancer-related fatigue and improves quality of life [33,34]. A preoperative exercise intervention improves patients’ functional capacity and thus may reduce perioperative morbidity [13]. These benefits make exercise interventions the backbone of current prehabilitation programs. The exact benefit of exercise depends on its type. There is no consensus on the optimal exercise regimen, which most likely explains the diversity of interventions seen throughout the literature.

Most available studies on EGC patients investigated unimodal prehabilitation consisting of exercise interventions only [23,24,26,27,28,30,32]. It is not surprising that the majority focused on preoperative inspiratory muscle training (IMT) because pulmonary complications are the most common after EGC surgery, affecting up to 20–40% of patients [35,36]. Pulmonary morbidity contributes to a prolonged hospital stay, increased treatment costs, mortality, and long-term impaired outcomes [9,37,38]. Thus, even the slightest improvement in these complication rates may significantly improve EGC treatment outcomes [9]. Studies by Dettling et al. [28], Valkenet et al. [23], and Argudo et al. [29] investigated IMT for 2–5 weeks using specialized inspiratory-threshold loading devices. These studies consistently showed the feasibility and safety of such prehabilitation [23,28,29]. Preoperative IMT improved inspiratory muscle function [23,28,29], but had no impact on postoperative morbidity [23,28]. However, the effectiveness of preoperative IMT with a special device may depend on the type of exercise. Adrichem et al. compared two different exercise protocols—high intensity and endurance IMT using Respifit S and Threshold-IMT devices, respectively. Both training protocols significantly increased maximal inspiratory pressure, representing an inspiratory function, but only high-intensity training decreased postoperative pulmonary morbidity [24]. Alternatively, preoperative respiratory rehabilitation can be conducted without any special equipment [26]. Yamana et al. demonstrated that even a short (>7 days) but intensive and complex supervised respiratory prehabilitation program consisting of different exercises for respiratory muscles together with aerobic exercise effectively reduces postoperative pulmonary morbidity in esophageal cancer patients [26].

Other types of exercise interventions investigated in unimodal prehabilitation studies were aerobic and resistance training with or without exercises for IMT and stretching [27,30,32]. Such a combination has a strong rationale because different exercises have different benefits. Aerobic exercises improve physical fitness and cardiac, respiratory, and musculoskeletal function even after a short training time (2–3 weeks) [39]. Resistance training promotes skeletal muscles hypertrophy, increases muscle mass, strength and function, and thus counteracts sarcopenia [40,41]. Resistance training is important in all age groups, including elderly and frail patients [40,41], who are at the highest risk for postoperative complications after EGC resections [42,43]. Unimodal exercise prehabilitation consisting of aerobic and resistance training is safe and feasible. It positively impacts fitness level, strength, and quality of life in EGC patients [30,44]. Moreover, a small matched-pair study from Japan suggested that such prehabilitation reduces the overall postoperative morbidity rate in high-risk patients undergoing gastrectomy [32]. Aerobic and/or resistance training is also the core intervention of multimodal prehabilitation programs [21,22,25,31]. Xu et al. showed that even the simplest aerobic exercise, such as walking, has a positive effect [25]. Only 25 min of nurse-supervised walking three times a week attenuates neoadjuvant chemoradiotherapy-induced decline in physical fitness and increases walking distance and hand-grip strength [25]. Similar benefits of aerobic and resistance training have been shown in other studies [21,22,31,32]. Despite notable differences between exercise protocols, all studies consistently showed positive effects by improved physical fitness levels [22,31], muscle mass [31], cardiorespiratory function [21], and reduced number of postoperative complications [32].

### 3.2. Nutritional and Psychological Interventions as Components of Multimodal Prehabilitation

Malnutrition affects about 80% of EGC patients and greatly negatively impacts treatment outcomes [45,46,47]. It increases the risk of systemic treatment-related toxicity, poor treatment adherence, postoperative morbidity, and mortality [48,49,50,51]. The etiology of malnutrition and the reasons for such a high incidence are multifactorial. It includes a variety of mechanisms related to cancer itself, the host response to the disease, and treatment [52]. First, tumors within the esophagus or stomach may simply cause a mechanical obstruction that prevents the passage of food through the gastrointestinal tract [48]. Second, cancer induces metabolic disturbances, immune system response, and CNS alterations that result in taste change, food aversion, and inhibition of absorption/digestion of nutrients [52,53]. Third, psychological stress, a common fear, depression, and anxiety, may also negatively impact appetite and food intake [52]. These changes result in insufficient caloric intake and promote depletion of micro-and macro-nutrients reserves in the body [53]. Moreover, cancer induces catabolic activities that lead to nutritional overconsumption and ultimately clinically relevant malnutrition [53]. Malnutrition is a modifiable risk factor, which can be efficiently adjusted if diagnosed early [54]. Well-timed nutritional interventions before major gastrointestinal surgery effectively improve nutritional status and quality of life and even reduce postoperative morbidity [55,56,57]. Thus, nutritional interventions seem like a necessary component of multimodal prehabilitation programs in EGC patients.

Currently, 5 studies investigated the effect of nutritional interventions that included food-based dietary advice ± oral nutritional supplements or enteral nutrition via feeding tubes if necessary [21,22,25,29,31]. Three of these studies showed an obvious positive effect of nutritional support by increased protein intake and a higher number of consumed calories [31]. Additionally, nutritional support attenuated neoadjuvant treatment-induced weight and muscle mass loss [21,25]. The other two studies did not measure outcomes that would directly represent nutritional interventions’ effect. Although, these studies showed that multimodal prehabilitation that includes nutritional support effectively improves the functional capacity and quality of life of EGC patients [22,29].

Besides physiological challenges, such as previously mentioned physical and nutritional issues, many EGC patients suffer from psychological and emotional distress [58,59,60,61,62]. Depression and anxiety impair compliance to cancer treatment and quality of life [58,63] and promote the development and progression of the disease. The proposed molecular mechanism for depression-induced carcinogenesis includes disease-related overproduction of reactive oxygen species leading to oxidative stress that promotes activation of different proto-oncogenes contributing to subsequent cancer development [62,64]. Therefore, it is not surprising that psychological distress is related to impaired long-term outcomes in cancer patients [58,65]. Psychological prehabilitation is suggested as a strategy to alleviate psychological distress and improve treatment outcomes. The systematic review by Tsimopoulou et al. summarized evidence from seven studies investigating psychological interventions before surgery for the breast, prostate, and colorectal cancer patients [66]. These interventions did not improve traditional surgical outcomes (postoperative morbidity and mortality or hospitalization time). Still, they positively affected patients’ reported outcomes, including psychological well-being, quality of life, and somatic symptoms [66]. In a cohort of EGC patients, only Allen et al. investigated psychological intervention as a part of multimodal prehabilitation [21]. The intervention consisted of six sessions of medical coaching to discuss health status, strength recognition, resilience profiling and development, social and support systems, emotional management, and goal setting [21]. The authors discuss that it may have contributed to higher neoadjuvant therapy completion rates by increasing patients’ resilience to their neoadjuvant journey. Nonetheless, it is difficult to reliably evaluate the impact of psychological support because the study had no clear endpoints for it [21].

## 4. Important Questions for the Wider Implementation of Prehabilitation Programs in Modern Esophagogastric Cancer Surgery and Gaps in Current Knowledge

This review summarized the current evidence for prehabilitation in modern EGC surgery. The available studies are very heterogeneous in design, type of interventions, and measured outcomes. All of them confirmed at least some positive effects of prehabilitation in terms of improved physical performance, nutritional status, quality of life, or even reduced number of postoperative complications [22,23,24,25,26,27,28,29,30,31,32]. Despite extensive evidence that supports the concept of prehabilitation, the heterogeneity of available studies prevents the standardization and wide adoption of the strategy. Clinicians willing to implement prehabilitation for EGC surgery will face several important questions, although not all can be answered yet.

### 4.1. Question 1: Multimodal or Unimodal Prehabilitation?

The most optimal regimen of prehabilitation remains unknown. Currently, multimodal and unimodal prehabilitation programs are available [67], with a similar level of evidence for effectiveness. Considering that EGC patients face physical, nutritional, and psychological challenges [68,69,70], multimodal prehabilitation may have greater benefits [67]. Multimodal prehabilitation requires more resources from healthcare professionals to train appropriate exercise interventions and provide nutritional and psychological support. Several ongoing trials investigating multimodal prehabilitation before EGC resection will elucidate the current unclarities in the topic [4,71,72].

### 4.2. Question 2: Supervised or Home-Based Prehabilitation?

Prehabilitation can be utilized in a hospital under the supervision of healthcare professionals or at home after initial training. Both strategies have advantages and disadvantages. On the one hand, supervised prehabilitation allows strict monitoring of the adherence to the program, and necessary adjustments are easy to make. Some conflicting evidence shows better outcomes of supervised training in patients with chronic low back pain [73], intermittent claudication [74], recent myocardial infarction [75], or after anterior cruciate ligament reconstruction [76]. However, the need for regular visits to treatment centers may preclude prehabilitation in patients who suffer logistical challenges. Additionally, additional visits to the hospital may be undesired by patients, especially in light of the ongoing COVID-19 pandemic. Tele-prehabilitation may be an alternative to supervised prehabilitation without traveling [30]. However, it remains unclear if supervised prehabilitation has any benefits over home-based prehabilitation [77,78]. Current literature indicates that the patient’s preferred method is home-based intervention; thus, a high level of adherence can be expected [79]. It seems that home-based unsupervised or semisupervised prehabilitation may be the most reasonable option for the majority of EGC patients.

### 4.3. Question 3: How to Ensure Adherence to Prehabilitation Program?

Insufficient adherence is among the biggest challenges limiting the effectiveness of prehabilitation [80]. Thus, there is a need for tools that would overcome the issue. Direct supervision by healthcare professionals could enhance a patient’s motivation and willpower to participate [81]. However, as mentioned previously, supervised prehabilitation has some major disadvantages. Incorporating behavioral science professionals’ support may improve patients’ motivation for interventions and adherence to prehabilitation [82]. However, only one [21] included psychological support among the available studies. Thus, stronger evidence is necessary, and future studies should elucidate the role of these specialists. Additionally, there is a need for studies to identify exact reasons precluding adherence to prehabilitation. Identification of barriers will let us create strategies to overcome them.

### 4.4. Question 4: At Which Stage of Treatment Should Prehabilitation Be Initiated?

The time frame between diagnosis and surgery is relatively short; thus, prehabilitation should be initiated as early as possible in patients undergoing surgery first. However, it is trickier with patients who need neoadjuvant therapy. One window for prehabilitation is the time between the completion of neoadjuvant therapy and surgery, which typically lasts at least 4–6 weeks [8]. Alternatively, prehabilitation may be initiated earlier, even at the time of diagnosis, and utilized throughout the neoadjuvant therapy. The feasibility of prehabilitation interventions in EGC patients undergoing cytotoxic neoadjuvant treatment has already been shown [21,25]. Early initiated prehabilitation may counteract some negative impacts of neoadjuvant treatment, including a decline in cardiorespiratory function and physical capacity [41,83]. These are major risk factors for morbidity in EGC surgery [84]; thus, it seems rational to schedule patients for prehabilitation at an early phase of the treatment.

### 4.5. Question 5: What Benefits of Prehabilitation Could Be Expected in Esophagogastric Cancer Patients?

#### 4.5.1. Prehabilitation’s Impact on Postoperative Morbidity

Three of seven studies investigating the impact of prehabilitation on postoperative morbidity after EGC resections showed a significant positive impact [21,22,23,26,28,39]. Two studies demonstrated that respiratory prehabilitation could reduce postoperative pulmonary complication rates [24,26]. One study showed aerobic- and resistance training-based prehabilitation significantly reduces postoperative morbidity after gastrectomy in high-risk patients with metabolic syndrome [32].

#### 4.5.2. Prehabilitation’s Impact on Adherence to Neoadjuvant Treatment Protocol

Two studies evaluated multimodal prehabilitation’s impact on adherence to all planned neoadjuvant treatments and showed conflicting results [21,22]. A randomized control study by Minella et al. showed a similar low (8%) non-compliance rate in the control and prehabilitation groups [22], while a slightly larger study by Allen et al. showed very different results [21]. A much higher non-compliance rate of 54% was observed in the control group, and prehabilitation significantly decreased it to 25% [21].

#### 4.5.3. Prehabilitation Impact on Quality of Life

Five studies investigated prehabilitation’s impact on quality of life [21,23,27,29,30]. Valkenet et al. showed that isolated inspiratory muscle training has no impact on quality of life-related outcomes [23]. In contrast, four studies that used complex exercise interventions demonstrated the positive effect of prehabilitation on social role functions [29], physical and emotional well-being [27,30], fatigue [29,30], anxiety and depression [30], and other quality of life-related outcomes [21,27,29,30].

#### 4.5.4. Prehabilitation Impact on Long-Term Outcomes

There is evidence that prehabilitation improves long-term outcomes in colorectal cancer patients [85]. However, its impact on long-term outcomes in EGC patients remains unknown. Future studies are necessary to address this question.

## 5. Conclusions

Prehabilitation has emerged as a novel strategy to optimize a patient’s status before major surgery. In this comprehensive review, we summarized the current evidence for the role of prehabilitation in modern EGC surgery. Despite the heterogeneity of the studies’ designs, all of them confirmed at least some positive effects of prehabilitation. The benefits included improved physical performance, nutritional status, quality of life, and even fewer postoperative complications. Future studies are necessary to determine the most optimal design of prehabilitation programs for esophagogastric resection.

## Figures and Tables

**Table 1:** Characteristics of studies investigating prehabilitation for esophagogastric cancer surgery.

Author; Year	Design	Description and Number of Participants; (n)	Measured Outcomes	N–O Score	Jadad Score
Allen et al. [21]; 2021	RCT	Esophagogastric cancer patients scheduled for surgery after neoadjuvant chemotherapy; (*n* = 54)	Primary outcome: Change in AT by CPET. Secondary outcomes: Change in peak VO2 by CPET; Sarcopenia measured by computed tomography; HGS; Health-related quality of life by EORTC QLQ-C30 questionnaire, Beck Anxiety Inventory, and Beck Depression score; Full adherence to the planned neoadjuvant chemotherapy and its toxicity; Weekly step count; Postoperative morbidity; 30-day hospital readmission rate; 3-year mortality rate.	N/A	3
Minnella et al. [22]; 2018	RCT	Esophagogastric cancer patients scheduled for surgery ± neoadjuvant treatment; (*n* = 68)	Primary outcome: Change in functional capacity over time by 6MWD. Secondary outcomes: Postoperative morbidity at 30 days; Length of hospital stay; 30-day hospital visits; 30-day readmission rates; 30-day death rates; Full adherence to the planned neoadjuvant chemotherapy; Compliance with prehabilitation program.	N/A	3
Valkenet et al. [23]; 2018	RCT	Esophageal cancer patients scheduled for surgery ± neoadjuvant treatment; (*n* = 270)	Primary outcome: Postoperative pneumonia rate. Secondary outcomes: Respiratory muscle function: maximum inspiratory pressure and inspiratory muscle endurance; Pulmonary function: expiratory volume in 1 s and FVC; Postoperative complication rate; Duration of mechanical bowel ventilation; Length of hospital stay; Quality of life by EuroQol-5D and SF-12 questionnaires; Physical activity by SQUASH questionnaire; Fatigue by MFI-20 questionnaire.	N/A	3
van Adrichem et al. [24]; 2014	RCT	Esophageal cancer patients scheduled for surgery ± neoadjuvant CRT; (*n* = 45)	Primary outcome: Postoperative pulmonary complications rate. Secondary outcomes: Length of stay; Stay in ICU; Number of reintubations; Maximal inspiratory pressure before and after training; Lung functions (FVC, FEV1, FEV1/FVC, and PIF); Feasibility by the number of IMT-related adverse events, compliance to training, and a self-estimated load of participation.	N/A	3
Xu et al. [25]; 2015	Pilot study (RCT)	Esophageal cancer patients scheduled for neoadjuvant CRT and surgery; (*n* = 59)	Primary outcomes: Functional walking capacity by 6MWD and strength by HGS; Nutritional status by BW and fat-free lean mass by bioelectrical impedance. Secondary outcome: Treatment tolerance by interruptions in chemotherapy or radiotherapy; unplanned hospital admission; grade > 2 neutropenia; fever > 38.5 °C; intravenous nutritional support and wheelchair use.	N/A	3
Yamana et al. [26]; 2015	RCT	Esophageal cancer patients scheduled for surgery ± neoadjuvant treatment; (*n* = 63)	Primary outcome: Postoperative pulmonary complication rate. Secondary outcomes: Respiratory function by FVC, FEV1, FEV1%, and PEF.	N/A	3
Christensen et al. [27]; 2018	Non-randomized control trial	Patients with GOJ adenocarcinoma scheduled for neoadjuvant treatment and surgery; (*n* = 50)	Primary outcome: Frequency of serious adverse events (defined as events that prevented surgery). Secondary outcomes: Neoadjuvant treatment tolerability; Postoperative complication rate; Postoperative hospital stay; Patient-reported tolerability to neoadjuvant treatment by FACT-E questionnaire; Response to treatment by infiltration of the resection margin and immunoscore, tumor regression grade by Mandard, and pathological tumor stage (pTNM).	8	N/A
Dettling et al. [28]; 2013	Non-randomized controlled trial	Patients scheduled for esophagectomy ± neoadjuvant treatment; (*n* = 83)	Primary outcomes: Feasibility by the occurrence of adverse effects, patients’ satisfaction; Initial effectiveness by pre-operative improvement in respiratory function. Secondary outcomes: Postoperative pneumonia rate; Length of hospital stay; Duration of mechanical ventilation; Reintubation rate; Length of stay in the ICU; Postoperative morbidity rate.	8	N/A
Argudo et al. [29]; 2020	Pilot study (prospective interventional study)	Esophagogastric cancer patients scheduled for neoadjuvant treatment and surgery; (*n* = 40)	Feasibility by TELOS components; Tolerability; Exercise capacity by cardiopulmonary exercise testing; Pulmonary and muscle function; Peripheral muscle function; Health-related quality of life by EORTC QLQ-C30 questionnaire.	6	N/A
Piraux et al. [30]; 2020	Pilot study (prospective interventional study)	Esophagogastric cancer patients scheduled for surgery ± neoadjuvant treatment; (*n* = 23)	Primary outcome Feasibility (recruitment, retention and attendance rates, adverse events, and patient satisfaction). Secondary outcomes Functional exercise capacity by 6MWD; CRF by FACIT-F scale; Quality of life by FACT-G questionnaire; Anxiety and depression by HADS questionnaire.	6	N/A
Yamamoto et al. [31]; 2016	Pilot study (prospective interventional study)	Gastric cancer patients aged ≥ 65 years with a diagnosis of sarcopenia scheduled for gastrectomy; (*n* = 22)	Nutritional intake (total number of calories and protein daily intake); Body composition (body mass, fat mass, lean body mass); Sarcopenia parameters (handgrip strength, gait speed, and skeletal muscle mass index).	6	N/A
Cho et al. [32]; 2014	Matched pair analysis	Patients with clinical stage I gastric cancer and metabolic syndrome scheduled for gastrectomy; (*n* = 72)	Primary outcome: Postoperative complications rate. Secondary outcomes: The operative time; Intraoperative blood loss; Hospital stay; Visceral fat and body weight.	7	N/A

RCT: randomized controlled trial; CRT: chemoradiotherapy; N/A: not applicable; GOJ: gastroesophageal junction; AT: anaerobic threshold; CPET: cardiopulmonary exercise testing; 6MWD: six minute walking distance; HGS: hand-grip strength; BW: body weight; FVC: forced vital capacity; FEV1: forced expiratory volume in the first second; FEV1%: forced expiratory volume in the first second predicted; PEF: peak expiratory flow.

**Table 2:** Structure of interventions in prehabilitation programs for esophagogastric cancer surgery.

Author; Year	Prehabilitation Group			Control Group
	Type of Prehabilitation (Unimodal vs. Multimodal)	Timing of Prehabilitation	Interventions Used for Prehabilitation	
Allen et al. [21]; 2021	Multimodal	Prehabilitation was initiated for 15 preoperative weeks.	Exercise intervention: supervised aerobic, resistance, and flexibility training twice a week and home-based exercise training three times per week; Nutritional intervention: needs-based nutritional interventions with frequent, tailored dietetic input from specialist dieticians, increasing calorie and protein intake where appropriate depending on assessments and physical activity levels; Psychological intervention: six sessions of medical coaching, which included discussion of health status, strength recognition, resilience profiling and development, social and support systems, emotional management, and goal setting.	Standard of care
Minnella et al. [17]; 2018	Multimodal	Prehabilitation was initiated before the initial surgery or at the time of neoadjuvant therapy.	Exercise intervention: individualized, home-based exercise training program including aerobic and strengthening exercise; Nutritional intervention: metabolic requirement was adjusted to meet the increased nutritional demand. Food-based dietary advice was given, and a whey protein supplement was prescribed to guarantee a daily protein intake.	Standard of care
Valkenet et al. [18]; 2018	Unimodal	Prehabilitation was initiated for 2 weeks or longer. When neoadjuvant therapy was administered, prehabilitation started afterward.	Exercise intervention: inspiratory muscle training.	Standard of care
van Adrichem et al. [19]; 2014	Unimodal	Prehabilitation was initiated for 3 weeks. When neoadjuvant therapy was administered, prehabilitation started afterward.	Exercise intervention: high-intensity inspiratory muscle training.	Exercise intervention: endurance inspiratory muscle training
Xu et al. [24]; 2015	Multimodal	Prehabilitation was initiated for 4–5 weeks during the neoadjuvant chemoradiotherapy.	Exercise intervention: nurse-supervised walking; Nutritional intervention: nutritional advice.	Standard of care
Yamana et al. [20]; 2015	Unimodal	Prehabilitation was initiated for ≥7 days before the surgery.	Exercise intervention: respiratory muscle training; muscle strengthening exercises for the lower limbs and abdominal muscles; biking on an ergometer.	Standard of care
Christensen et al. [25]; 2018	Unimodal	Prehabilitation was initiated at the time of neoadjuvant treatment.	Exercise intervention: supervised high-intensity aerobic and resistance exercise.	Standard of care
Dettling et al. [26]; 2013	Unimodal	Prehabilitation was initiated for 2 weeks or longer.	Exercise intervention: inspiratory muscle training.	Standard of care
Argudo et al. [21]; 2020	Multimodal	Prehabilitation was initiated after neoadjuvant chemotherapy for 5 weeks.	Exercise intervention: high-intensity interval training on the ergometric bicycle; respiratory muscle training using a respiratory muscle trainer. Nutritional intervention: individualized nutritional therapy based on nutritional status and ability to fulfill caloric-protein requirements.	N/A
Piraux et al. [22]; 2020	Unimodal	Prehabilitation was initiated for 2–4 weeks before the surgery.	Exercise intervention: aerobic, resistance, and respiratory muscle training using an online tele-prehabilitation platform.	N/A
Yamamoto et al. [23]; 2016	Multimodal	Prehabilitation was initiated for 3 weeks, although the actual duration differed depending on the surgery date.	Exercise intervention: handgrip training, walking, and resistance training; Nutritional intervention: nutritional advice and 2.4 g daily oral supplementation with leucine metabolite b-hydroxy-b-methylbutyrate (HMB).	N/A
Cho et al. [27]; 2014	Unimodal	Prehabilitation was initiated for 4 weeks.	Exercise intervention: aerobic exercise, resistance training, and stretching.	Standard of care

CRT: chemoradiotherapy; N/A: not applicable.

**Table 3:** Outcomes of included studies evaluating prehabilitation for esophagogastric cancer surgery.

Author; Year	Prehabilitation Impact on Physical Status	Prehabilitation Impact on Postoperative Outcomes	Other Effects of Prehabilitation
Allen et al. [21]; 2021	Prehabilitation attenuated peak VO2 decrease and skeletal muscle loss following neoadjuvant therapy. Additionally, HGS was better retained in the prehabilitation group, and patients in this group were more physically active by higher weekly step count.	Prehabilitation had no impact on the number and severity of complications, length of hospital stay, 30-day readmission rates, and 3-year cancer-related mortality.	Prehabilitation improved QoL by global health status after 2 cycles of neoadjuvant chemotherapy and at 2 weeks, 6 weeks, and 6 months postoperatively. Additionally, prehabilitation resulted in better BAI and DBI II scores preoperatively and 6 weeks and 6 months postoperatively. A higher proportion of patients in the prehabilitation group received neoadjuvant chemotherapy at full dose.
Minnella et al. [17]; 2018	Prehabilitation improved functional capacity before and after surgery by increasing 6MWD.	Prehabilitation had no impact on the number and severity of complications, length of hospital stay, emergency department visits, and readmission rates.	N/A
Valkenet et al. [18]; 2018	Prehabilitation resulted in a higher increase in inspiratory muscle strength and endurance.	Prehabilitation did not affect postoperative pneumonia and other postoperative complication rates.	Prehabilitation did not affect the quality of life, fatigue, and physical activity levels.
van Adrichem et al. [19]; 2014	The increase in maximal inspiratory pressure was similar between the groups which received preoperative inspiratory muscle training.	The incidence of postoperative pulmonary complications, length of stay, and the number of reintubations were lower in the high-intensity inspiratory muscle training group.	N/A
Xu et al. [24]; 2015	Prehabilitation ameliorated decline in 6MWD and hand-grip strength.	N/A	Prehabilitation ameliorated weight and lean muscle mass loss. Additionally, patients in the prehabilitation group had a significantly lower need for intravenous nutritional support and wheelchair use.
Yamana et al. [20]; 2015	Prehabilitation did not affect respiratory function representing parameters (FVC, FEV1, FEV1%, and PEF).	Prehabilitation ameliorated the severity of postoperative complications (by lower Clavien–Dindo score) and postoperative pneumonia (by lower Utrecht Pneumonia Scoring System score).	N/A
Christensen et al. [25]; 2018	Prehabilitation resulted in improved fitness and muscle strength.	Prehabilitation did not affect the postoperative complication rate.	Prehabilitation resulted in improved quality of life by FACT-E score.
Dettling et al. [26]; 2013	Prehabilitation increased inspiratory muscle strength and endurance.	Prehabilitation did not affect postoperative pneumonia and other complication rates.	N/A
Argudo et al. [21]; 2020	Prehabilitation improved exercise capacity in terms of VO2 peak and workload and distance improvement in the 6MWD and inspiratory and expiratory muscle strength.	N/A	Prehabilitation resulted in the improvement of some domains of health-related quality of life (social and role functions).
Piraux et al. [22]; 2020	N/A	N/A	Prehabilitation improved fatigue, quality of life, physical well-being, emotional well-being, and anxiety.
Yamamoto et al. [23]; 2016	Prehabilitation significantly increased handgrip strength.	N/A	Prehabilitation improved nutritional uptake by increasing calorie and protein intake.
Cho et al. [27]; 2014	N/A	Prehabilitation decreased hospital stay and the number of severe postoperative complications (anastomotic leakage, pancreatic fistula, intra-abdominal abscess, and other severe abdominal complications).	Prehabilitation significantly decreased BMI, bodyweight, abdominal circumference, and visceral fat.

6MWD: six minute walking distance; N/A: not applicable; FVC: forced vital capacity; FEV1: forced expiratory volume in the first second; FEV1%: forced expiratory volume in the first second predicted; PEF: peak expiratory flow.
